# Mapping Scandinavian Hospital‐at‐Home Organisations and Interventions for Patients With Acute Somatic Illness—A Scoping Review

**DOI:** 10.1111/scs.70312

**Published:** 2026-09-07

**Authors:** Kristina Kock Hansen, Dorthe Eg Holm, Maria Klitgaard Christensen, Peter Biesenbach, Kristian Kidholm, Christian Backer Mogensen, Jette Holt, Pia Lysdal Veje, Mette Elkjær, Caroline Moos

**Affiliations:** ^1^ Research Center for Integrated Healthcare Region of Southern Denmark Aabenraa Denmark; ^2^ Research Unit of General Practice, Institute of Public Health University of Southern Denmark Odense Denmark; ^3^ Department of Applied Health Science University College Southern Denmark Kolding and Aabenraa Denmark; ^4^ Department of Clinical Research, Faculty of Health Sciences University of Southern Denmark Odense Denmark; ^5^ Department of Emergency Medicine University Hospital of Southern Denmark Esbjerg Denmark; ^6^ Center for Innovative Medical Technology Odense University Hospital and University of Southern Denmark Odense Denmark; ^7^ Department of Emergency Medicine University Hospital of Southern Denmark Aabenraa Denmark; ^8^ Department of Regional Health Research University of Southern Denmark Odense Denmark; ^9^ Infectious Disease Epidemiology & Prevention, The National Center for Infection Control (CEI) Copenhagen Denmark; ^10^ Department of Clinical Research University Hospital of Southern Denmark Aabenraa Denmark

**Keywords:** acute illness, hospital‐at‐home, hospital‐based home care services, intersectoral collaboration, literature review, Scandinavia, scoping review

## Abstract

**Introduction:**

Hospital‐at‐Home (HaH) has been implemented in several countries. However, HaH interventions vary depending on organisational structures and target populations. In Denmark, Norway, and Sweden, HaH is offered in selected regions and municipalities, often as pilot or quality projects. Currently, there are no guidelines or systematic overviews of Scandinavian HaH models.

**Aim:**

This review aims to map published research that describes Scandinavian HaH organisations, interventions, and patient characteristics for acute somatic illness.

**Methods:**

In this scoping review, papers were identified through a systematic search of Medline, Embase, and CENTRAL, supplemented by a grey literature search. Eligible studies focused on Scandinavian adults (≥ 18 years) with acute somatic illness requiring initial contact with an emergency department (< 24 h), out‐of‐hours doctor, or a general practitioner. Exclusions criteria were early discharge care, post‐discharge care, self‐monitoring or single health care contacts. Two researchers screened studies in a two‐step process.

**Results:**

Ten studies met the inclusion criteria. Three primary HaH models were identified: (1) municipal acute nursing teams collaborating with general practitioners (GPs) and hospital physicians or municipal mobile acute care with physicians, (2) hospital‐affiliated emergency department (ED) teams providing mobile acute care, and (3) specialised acute units integrating virtual and in‐person health care. Common interventions included intravenous therapy, point‐of‐care diagnostics, clinical monitoring and digital consultations. The majority of HaH patients were older adults with multiple chronic conditions, commonly treated for infections, dehydration, or exacerbations of chronic diseases.

**Conclusion:**

Scandinavian HaH can be categorised into three models. The primary distinction between the models lies in the organisational affiliation of the nursing teams, which plays a pivotal role in defining the care structure.

## 1 Introduction

Hospital‐at‐Home (HaH) is shown to improve patient satisfaction, reduce stress associated with hospitalisation and ease the burden on hospitals [1, 2, 3, 4, 5]. Health care systems worldwide are under increasing pressure, with unprecedented numbers of people reaching retirement and living longer with multiple chronic illnesses [6, 7]. Additionally, challenges in retaining and recruiting health care professionals worsen the situation. Scandinavian governments are responding to demographic challenges by reorganising health care systems to optimise resource use. One key strategy is Hospital‐at‐Home (HaH) that aims to reduce hospital admissions and ease pressure on acute care [8].

HaH is defined as an acute clinical service that serves as a substitute for inpatient hospital care [9]. There are two main approaches within HaH—*Admission avoidance* and *early discharge* [10, 11]. Admission avoidance prevents hospitalisation entirely, while early discharge enables patients to return home earlier to receive short‐term acute or sub‐acute care. HaH literature describes services catering to a broad spectrum of patient needs, including end‐of‐life care, early discharge support, and even admission avoidance [7, 9]. Four Cochrane reviews have focused on the effects of HaH with varying interventions and patient populations [11, 12, 13, 14]. Admission avoidance appears to be an adopted alternative to hospitalisation for adults with chronic obstructive pulmonary disease (COPD) exacerbations [12] or palliative care requirements [13]. Research has suggested that avoiding admission by providing acute care in the home is equally efficient to hospital care for older patients with acute cardiac failure [15] or oncological populations [16]. Generally, studies about HaH reduce patient stress, shorten the length of hospital stay, and lower the cost of care [5]. The type of health care professionals delivering HaH varies significantly, reflecting the diverse nature of these programmes.

Despite growing interest and implementation of HaH in Scandinavia, there appears to be no general overview of this model of care for acute illness [17]. Key information on organisations, interventions, and patient characteristics remains limited [7, 18, 19]. A newly published systematic review on HaH implementation highlights the need for clear eligibility criteria, a trained workforce, and effective communication between staff, patients, and caregivers to ensure success [20]. Given the similarities across Scandinavian countries in terms of health care organisation, economy, and demographics, a comparative overview could offer valuable insights. A scoping review is well‐suited to this study as it enables a broad mapping of the organisations, interventions, and patient populations of Scandinavian HaH, where existing evidence is diverse and scattered.

This scoping review aims to map published research that describes Scandinavian HaH organisations, interventions, and patient characteristics for acute somatic illness.

## 2 Methods

This scoping review was guided by the JBI (Johanna Briggs Institute) Manual for Evidence Synthesis [21] and reported according to the PRISMA‐ScR (Preferred Reporting Items for Systematic Reviews and Meta‐Analyses) guidelines [22]. The scoping review protocol was developed and uploaded to the Open Science Framework (DOI: 10.17605/OSF.IO/WKE7X) and subsequently peer‐reviewed and published providing a transparent, robust and valid foundation for the study [23]. For the purposes of this review, we define ‘intervention’ broadly to include processes of care such as diagnostic testing, drug delivery, and other therapeutic procedures.

### 2.1 Review Questions

How do Scandinavian HaH models organise care for acute somatic illness?

- What types of interventions are provided?
- What are the characteristics of patients considered eligible for acute care at home?

### 2.2 Literature Search

Searches were conducted on the 11th of August 2025 using the databases Medline (OVID), Embase (OVID), and CENTRAL (Cochrane Library). No time or language limits were applied to ensure a broad, unbiased search [24]. Grey literature searches were conducted in ClinicalTrials.gov and the official websites of Scandinavian health authorities or sectors to identify other possible relevant studies.

The search strategy was developed using the Population, Concept and Context (PCC) typology [25]. Three facets were included in the block search—Acute illness or care (population), Hospital‐at‐Home (concept) and Scandinavia (context). Each facet used standardised keywords from the database's controlled vocabulary thesaurus and free text words combined with the Boolean operator ‘OR’. The Hospital‐at‐Home facet was inspired by Cochrane reviews that developed search strings for this concept, albeit in two different reviews [10, 11]. After each facet was developed and tested, the search strategies for the three facets were combined using the Boolean operator ‘AND’. Forward and backward citation searches were conducted in Web of Science to identify additional studies either cited by, or citing, the included articles, ensuring a comprehensive capture of relevant literature. The search strategy was developed in collaboration with a university librarian specialised in literature search in the area of public health. The search string for all databases is available in Table S1. The results from the grey literature search were screened manually, with assistance from Google Translate. A detailed description of the search terms used for the grey literature search can be found in Table S2.

### 2.3 Inclusion and Exclusion Criteria

#### 2.3.1 Population

Studies were eligible if they described adults ≥ 18 years with the onset of an acute somatic illness requiring emergency care and initial contact with an emergency department (< 24 h), out‐of‐hours doctor or a general practitioner. Studies were excluded if they described adults requiring post‐surgery, acute psychiatric, long‐term, end‐of‐life or palliative care.

#### 2.3.2 Concept

Studies were included if interventions described admission avoidance to a hospital. HaH was defined as acute treatment at home or in a nursing home under the medical responsibility of a hospital or GP with a service that would typically require hospital admission. Studies were excluded if they focused on post‐discharge care, early discharge care, self‐administration, self‐monitoring, discharge to a temporary placement or a single contact with health care providers with no follow‐up.

#### 2.3.3 Context

Studies were included if the intervention was conducted in Scandinavia. Scandinavia was defined as Denmark, Norway, Sweden, Finland, Greenland, Iceland, and the Faroe Islands.

In Denmark, Norway, Sweden, and Finland, health care is primarily provided by the state through regions/counties, while municipalities play a role in primary health care. It is pertinent to note that in Finland municipality responsibility is limited to providing preventive services [26, 27, 28, 29]. Additionally, the organisation of GPs also varies across Scandinavia. In Norway and Finland, GPs operate under municipalities as the provider of primary health care. In Denmark and Sweden, GPs are contracted and accountable at a regional level. In contrast, health care is more centralised in Iceland, Greenland and the Faroe Islands, with municipalities playing a minimal role [30, 31, 32].

#### 2.3.4 Type of Sources

Publication type was restricted to peer‐reviewed studies to ensure research of reasonable quality but included all study designs.

### 2.4 Screening Process and Data Extraction

Results from the searches were imported into Covidence, a web‐based collaboration software platform that streamlines the production of systematic and other literature reviews [33]. Duplicates were eliminated, and results were screened independently by two researchers in a two‐step process—title/abstract screening and full‐text screening. Studies not qualifying for inclusion at full‐text screening were excluded with justification. Any disagreements were resolved through discussions to reach consensus.

Three researchers extracted data blinded using an adjusted template from Covidence. The data extracted included first author, publication year, country, research design, aim, number of patients, outcome measures, results, roles and responsibilities of health care professionals, task allocation and interprofessional agreements, intervention descriptions, patient characteristics (sex, age, comorbidities), included studies' inclusion criteria and reason for admission. Our focus in this review is on the roles and compositions of health care professionals in organisations as well as distribution of tasks across sectors and not on other components of HaH organisations such as data sharing and digitalisation, infrastructure, physical framework, etc.

### 2.5 Data Analysis

We mapped and synthesised data from the included studies to identify organisations, interventions and patient characteristics. Key themes guided the categorisation of organisational models. Data were extracted and are visualised in tables to show roles, responsibilities and cross‐sector collaborations.

Using an iterative approach, we refined the models as new insights emerged, comparing roles and relationships to highlight patterns and differences. Final organisational models were narratively described, visually illustrated and discussed in terms of strengths and weaknesses.

## 3 Results

### 3.1 Study Inclusion

**FIGURE 1:**
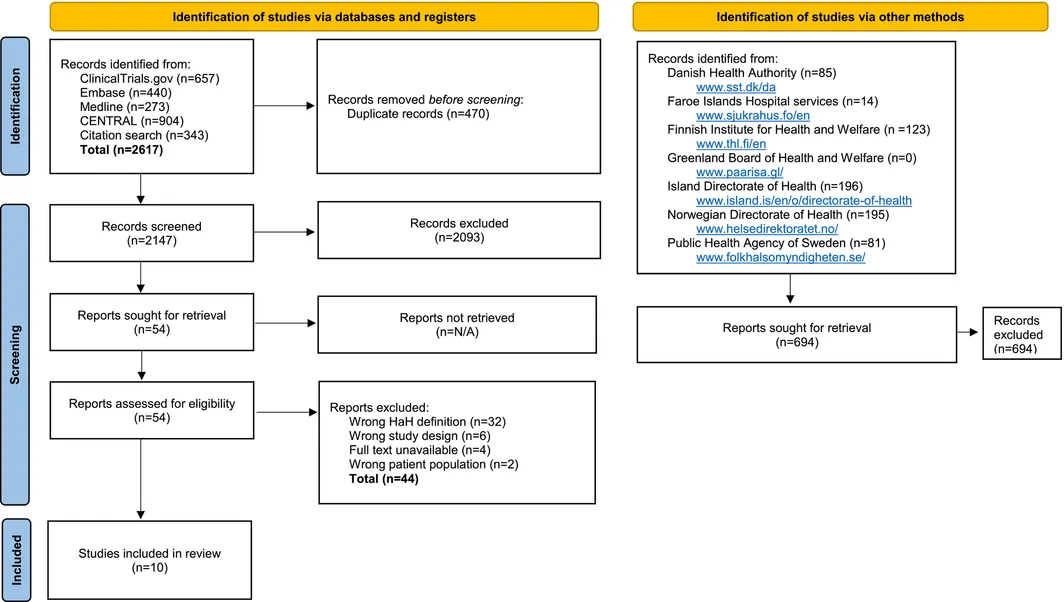
PRISMA 2020 flow diagram. This work is licensed under CC BY 4.0. To view a copy of this license, visit https://creativecommons.org/licenses/by/4.0/.

A total of 2147 studies were screened, resulting in 10 relevant studies consisting of qualitative and quantitative data. See Figure 1 for the PRISMA flow diagram. Most studies were conducted in Denmark (*n* = 6) [34, 35, 36, 37, 38, 39], with the remaining studies in Sweden (*n* = 4) [40, 41, 42, 43]. The studies were published between 2014 and 2025, with the majority published within the last four years. See Table 1 for study summary.

**TABLE 1:** Summary of the included studies (*n* = 10).

#	First author	Study design	Objectives	Number of participants	Inclusion criteria	Exclusion criteria	Outcome measures
	Year						
	Country						
1	Cramer 2014 Denmark	Quantitative registry‐based study	To identify patient characteristics for increased risk of 30 days rehospitalisation, after referral to HaH	379 patients	Treated with parenteral therapy	Patients < 18 years Without documentation on treatment in the register Without correct entered civil registry number	Primary: Readmission (30‐day follow‐up) Mortality (30‐day follow‐up) Secondary: Not described
2	Mogensen 2018 Denmark	Randomised, open‐labelled, multicenter parallel group clinical trial with two arms	Is the patient's own GP more effective than a hospital specialist at reducing hospital admissions without affecting the recovery or death rates?	131 patients	≥ 60 years old patients Residing in one of the four municipalities	Patients were assessed by others than their usual GP Permanently residing in a nursing home Unable to give written consent or had no relatives to give acting consent Municipality capacity fully utilised	Primary: Number of hospital admissions within 7 days Secondary: Number of admissions within 14, 21 and 30 days Deaths within 30 and 90 days Changes in performance tests
3	Udesen 2021 Denmark	Mixed‐method study	To describe patients treated by a municipal acute care team and to explore patients' and caregivers' experiences with at‐home treatment	3231 patients were treated by ATO 307 patients and 168 caregivers completed survey interviews	Only patients in need of short‐term treatment	Cognitive impairment Too ill Hospitalised	Primary: Patients' and caregivers' experience Secondary: Not described
4	Bove 2022 Denmark	Qualitative study	To investigate the experiences of Danish patients treated at home for an acute illness instead of being hospitalised	2286 patients were treated by TST 21 patients were interviewed	Patients treated at home by TST Willing to participate in interviews Speaking Danish or English Able to give informed consent	Not described	Primary: Patient satisfaction Secondary: Not described
5	Teske 2023 Sweden	Qualitative study	To describe the experiences of the mobile care in Sweden	12 participants with experience of mobile care (nurses, physicians, civil servants and politicians)	Not described	Not described	Primary: Experiences with mobile care Secondary: Not described
6	Kastengren 2024 Sweden	Single‐centre descriptive study	To evaluate Sweden's first implementation of a 24/7 high‐acuity virtual in‐patient ward through a DPIPC program	200 patients	Patients with acute medical illness requiring inpatient‐level care and were: Age ≥ 18 years Lived within 30 min from the hospital Concentrated to the DPIPC program Patient or relative could ambulate to the front door Had a set diagnosis and care plan Were clinically stable Would require no more than four planned home visits per day	Need of a higher care level than a regular inpatient ward New onset of oxygen > 3 L High risk of falling Inability to operate a tablet Home environment unsuitable for HaH	Primary: Patient satisfaction Secondary: Health care use Safety Quality
7	Smedemark 2024 Denmark	Prospective observational non‐randomised pilot and feasibility study	To investigate whether Ext‐POCT during in‐home assessments among older adults was feasible and to pilot‐test the study design including the intervention consisting of FLUS and in‐home analysis of biological material	100 patients	Adults ≥ 65 years Participants had at least one of the following inclusion criteria: Cough Dyspnea Fever Chest pain Fall Functional decline	Patients with known moderate to severe cognitive impairment	Primary: Feasibility of study design and extended point‐of‐care testing Secondary: The potential clinical impact of extended point‐of‐care testing
8	Thomsen 2024 Denmark	Study protocol for an open‐label randomised controlled trial with a 1:2 allocation ratio of standard hospital admission versus HaH treatment	To reduce 30‐day acute readmission after discharge and improve the quality of life of elderly acute patients	Data not yet available	Age ≥ 65 years Diagnosed with a stable acute medical conditions Living in own home or a nursing home Residing in one of the three municipalities Visited at home by the GP, out‐of‐hours medical service or an EMS physician Speaks and understands Danish Informed consent to participate in the study	Not being able to give written consent Municipality acute team capacity fully utilised	Primary: Rate of 30‐day acute readmission Quality of life Secondary: Basic functional mobility Resource use in health care Primary and secondary health care cost Incremental cost‐effectiveness ratio Mortality rate 3 months after discharge
9	Falchenberg 2024 Sweden	Qualitative multiple case study design	To explore the health care work of the METs when addressing patients' emergency care needs in their homes	26 patients were treated (MET A) 12 physicians and nurses were interviewed (MET A)	MET A: Patients aged > 18 and frail elderly who suffer from treatable conditions that have occurred suddenly	Not described	Primary: Focus on the METs reasoning and actions
10	Erwander 2025 Sweden	Quantitative pre‐post intervention study	To assess whether the mobile team intervention reduced ED visits and hospitalisations	2163 patients in the pre‐intervention 2197 patients in the post‐intervention	Patients ≥ 65 years receiving municipality home care	Not described	Primary: Number of ED visits and hospitalisations

Abbreviations: ATO, Acute Team Odense; DPIPC, digi‐physical inpatient care; ExtPOCT, Extended Point‐of‐Care Testing; FLUS, Focused Lung Ultrasound; GP, general practitioner; HaH, Hospital‐at‐Home; IQR, Interquartile range; LOS, length of stay; MET, Medical Emergency Team; MET A, Operating from a hospital‐affiliated Emergency Department; NPS, Net Promotor Score; TST, cross‐sectoral and interdisciplinary team.

### 3.2 Three Models Describing the Organisations in Scandinavian HaH Solutions

**FIGURE 2:**
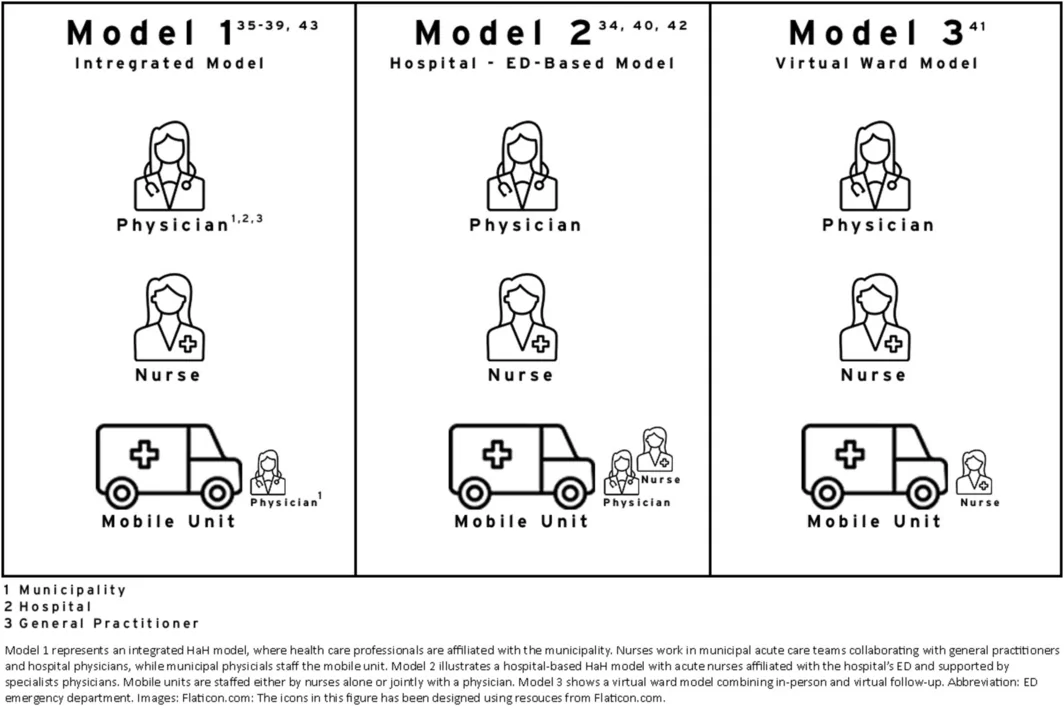
Illustration of three HaH models in Scandinavia.

An overview of the organisations, which in this review includes the roles and responsibility of health care professionals and agreements and tasks allocation between them, is illustrated in Table 2. In all the studies, specialised acute nursing teams treated patients at home. These specialised nursing teams were affiliated differently, and based on these organisational affiliations, the framework of different HaH organisations in Scandinavia can be illustrated with three models (Figure 2).

**TABLE 2:** Overview of organisations (*n* = 10).

#	Person with responsibility for treatment and referral	Health care professionals involved	Agreements and tasks allocated among health care professionals and across sectors (key findings/roles of health care professionals)	In which period is service and referral to HaH possible?
Cramer	Physician at the referring authority (GP or hospital physician)	9 specialised nurses from a municipality (HaH team) Physicians (local hospital) GP (emergency)	Not described	Between 7 a.m. and 11. p.m., 7 days a week (The HaH team endeavours to visit the patient's home within one hour after referral) Between 11 p.m. and 7 a.m. the regular primary care team manages the care and observations
Mogensen	The hospital specialist or the GP	All municipality nurses with a 1‐day training program GPs 8 physicians—all specialists in internal medicine	(1) The hospital arm: Assessed by hospital specialists (prescribe blood tests and imaging studies within 30 min after arrival) A detailed plan was sent electronically to the municipal HaH nurses (within 4 h) The patient was transported to the HaH destination together with medical supplies (2) The GP arm: The patients were either seen in their own GPs consultation or at home The GP examined the patient and performed a limited number of laboratory tests The GP prescribed a treatment and observation plan, which was immediately electronically available to the HaH nurses If the GP prescribed IV treatment, municipal services brought medicine and IV fluids to the patient	Referral through the GPs in their weekday opening hours The GP should be available the first two working days after inclusion (during daytime) Outside working hours, the local GP on duty could be consulted The hospital specialists could be contacted directly by telephone within 48 h from admission but was unable to visit the patient at home or at the nursing home (the HaH service only lasted up to 48 h after the inclusion)
Udesen	Various health care providers—most often by GP, municipal staff and hospital staff.	20 highly qualified specialised nurses GPs Municipal staff (nurses and nursing assistants) 3 departments at a University Hospital—ED, Geriatric Department, Palliative Team. The out‐of‐hours service The Emergency Medical Dispatch Centre	Not described	Not described
Bove	ED physician	10–15 nurses (TST) affiliated with an ED 3 physiotherapists (ED) 3 specialist physicians (ED)	Tasks are performed by the TST nurses supported by ED physicians or in close collaboration with nurses from the municipality All patients have been examined and assessed by a senior physician in the ED TST refers to the head nurse and chief physician in the ED	From 8:00 to 22:00, 7 days a week
Teske	The municipal nurse in home health care together with the home health care physician (GP)	Mobile care: Physicians (often specialists in geriatrics or internal medicine) Nurses (affiliated with hospital) GPs Nurses (municipality)	Joint home assessment by hospital physician and nurse. If possible, the municipal nurse must attend Option for urgent visits beyond scheduled visits Primary assessment by the municipal nurse, followed by contact to the mobile team Possibility of direct admission from the home to the hospital (nursing department)	The mobile teams usually start from a hospital and work office hours On evenings and weekends, the home care team takes over
Kastengren	DPIPC physician (clinicians/HaH physicians—specialists in internal medicine)	Hospital clinicians DPIPC Ambulating nurse or nurse practitioner A physiotherapist (available) Organised in an around‐the‐clock virtual medical command centre	Initiation of DPIPC from the hospital physician to the HaH physician Patients were introduced by a nurse to an in‐home technology kit consisting of a tablet (upon admission) The patient was transferred home by taxi service or by DPIPC personnel Daily digital video rounds by the DPIPC physician Introductory home visit within 2 h by an ambulating nurse or nurse practitioner (securing DPIPC is a possibility) Nurse prepared all ordered medication for the first 2 days of the program In the event of patient deterioration, virtual assessment and on‐site response was performed (in the home or at the hospital)	Patients could be admitted between 7 AM and 6 PM all days of the week At least one physical visit per day by ambulating nurse Nurse was available to the patients 24 h a day and could at any time contact the on‐call DPIPC physician for a consultation and/or visit the patient for an urgent evaluation
Smedemark	PCP	The ACHCS in a municipality PI—a hospital physician, certified and trained in thoracic ultrasound	The usual in‐home assessment was made by the ACNs after referral from PCPs or home care service personnel PI accompanied the ACNs during in‐home assessment and performed ExtPOCT Information is communicated to the PCP	Not described
Thomsen	ED physician	ED EMS GPs Acute nurses from 3 municipalities specialised and experienced in providing acute care at home	Patients will be identified through an emergency call by their referring physician (GP, out‐of‐hours medical service or EMS physician) The referring physician and the ED physician will jointly (through a conference call) evaluate, whether the patient can be included in the study and plan the treatment in case of inclusion The municipality acute team is called by the ED physician and the treatment is planned (either by phone or virtually on an iPad) The acute team contacts the ED physician if needed The ED physician may request a short ED check‐up to assess whether HaH can continue or hospital admission is needed	The acute team is available around the clock and visits patients whenever required under the supervision of the ED physician In case of emergency, the patient can call 112, and the EMS will visit the patient
Falchenberg	ED physician	Physicians (ED) Nurses (ED)	Nurses drove the vehicle and were responsible for checking the equipment, restocking supplies in the vehicle and carrying other equipment Physicians were responsible for bringing the laptop bag and ultrasound equipment Physicians conducted the examinations based on the ABCDE principles Most often, the nurse measured vital parameters or prepared the laboratory equipment during the ongoing examination Nurses were responsible for retrieving the laboratory equipment Physicians made decisions regarding treatment and discussed treatment options with patients and other health care personnel present The physician was responsible for documentation and contact with the receiving unit	MET A: 7.30 AM‐16 PM weekdays (physician and nurses)
Erwander	Municipal physician	MHC nurses 3 specialised municipal mobile team physicians Municipal on‐call physicians (out‐of‐hours)	Nurses conduct physical examinations Nurses initiate a pre‐established treatment plan (physician planned) Physicians develop care and treatment plans, provide consultation and conduct home visits on short notice Patients had multiple or single contacts (follow‐up by the GP at a primary health center) with the mobile team	Monday to Friday from 9.00–16.00

Abbreviations: ABCDE, airway, breathing, circulation, disability, and exposure; ACHCS, acute community health care services; ACNs, acute community nurses; DPIPC, digi‐physical inpatient care; ED, emergency department; EMS, emergency medical service; ExtPOCT, extended point‐of‐care testing; GP, general practitioner; HaH, Hospital‐at‐Home; MET A, Operating from a hospital‐affiliated Emergency Department; MHC, municipal home care; PCP, primary care physician; PI, primary investigator; TST, cross‐sectoral and interdisciplinary team.

Most HaH descriptions described a collaboration between the nursing teams, the GPs, the hospital physician (rarely specified which affiliation), or a combination of all three health care professionals. Patients were referred to HaH interventions from hospitals (often ED), GPs, health care professionals in the municipalities, and out‐of‐hours or emergency medical services (EMS) physicians. In most studies, duty of care rested with the GP or the emergency department (ED) physician.

In Model 1, representing an integrated HaH model, healthcare professionals were affiliated with the municipality. The HaH nurses were employed in municipal acute care teams [35, 36, 37, 38, 39, 43], communicating and collaborating with GPs, hospital physicians, or out‐of‐hours physicians. In addition, municipal physicians (as opposed to ‘ordinary’ GPs) staffed mobile units and provided HaH. Although follow‐up visits are not explicitly described in the specific study [43], correspondence with the first author confirmed that follow‐up was indeed conducted.

In model 2, an ED based model, the specialised nursing teams were affiliated with the hospital's ED [34, 40, 42] supported by the specialist physicians in the ED and physiotherapists. This type of HaH care was delivered by mobile units, either staffed solely by acute nurses, where the physician does not physically see the patient but is consulted, or where the nurses and the physician visit the patient together. The physicians described in this model were ED physicians, geriatrics specialists, or internal medicine specialists.

Model 3, a virtual ward model, featured a specialised acute unit consisting of physicians (specialists in internal medicine), registered nurses and nurse practitioners, which were extensions of an existing hospital department (emergency or medical) [41]. A physiotherapist was available if needed. The purpose of this unit was to visit patients, initiate treatment, and follow up with additional physical visits or most often provide a virtual solution for health care.

### 3.3 Interventions

Common HaH interventions included intravenous fluid therapy and medication administration (primarily antibiotics), blood tests via point‐of‐care testing, clinical observations and measurements of vital signs. However, 12‐lead electrocardiogram, ultrasound, bladder scans and blood transfusions were other types of treatment and diagnostics offered to patients as HaH interventions. A summary of the interventions is listed in Table 3.

**TABLE 3:** Overview of interventions (*n* = 10).

#	Treatments/Services
Cramer	Parenteral therapy (fluids and medication)
Mogensen	Administration of IV fluid and IV medicine Inhalation therapy Measurement of vital values Clinical examination and investigation Triage Urine stix
Udesen	Para‐clinical samples Intravenous antibiotic Fluid therapy
Bove	Intravenous fluids Medication administration, primarily antibiotics Blood tests Clinical observations Measurements such as vital signs, blood pressure, blood gasses, microbiology specimens
Teske	Intravenous treatment (diuretics, antibiotics, pain relief or blood transfusions)
Kastengren	Physical examination Vital signs monitoring Intravenous infusions (antibiotics, diuretics, fluids for rehydration) Wound care Medication administration Mobile diagnostics (ultrasound and 12‐lead ECG) Video calls (by physician) facilitated by an ambulating nurse
Smedemark	Clinical assessment by ACN (vital signs, capillary C‐reactive protein, haemoglobin) A physician performed: FLUS Venipuncture with bedside analysis (electrolytes, creatinine, white blood cell differential count) Nasopharyngeal swab Urine samples
Thomsen	Vital signs Venous blood samples ECG Bladder scan Intravenous medications
Falchenberg	Measurements (blood pressure, pulse, saturation, ear temperature) Monitoring Suturing and gluing wounds Urin drain, insert of urinary catheter or urin analysis Blood tests Ultrasound Intravenous and oral medications (antibiotics and pain reliever) Intravenous fluids Respiratory care with oxygen
Erwander	Treating leg ulcers Taking blood tests Managing medications Monitoring and regulating blood pressure Addressing heart failure and pulmonary disease

Abbreviations: ACN, acute community nurse; ECG, electrocardiogram; FLUS, focused lung ultrasound; IV, intravenous.

### 3.4 Patient Characteristics

The number of included patients ranged between 100 [37] and 3231 [39]. Five out of ten studies included information about median or mean age (see Table 4) and the majority of these studies described ≥ 80 years of age. Four studies described the degree of medical severity of the included patients using either the Clinical Frailty Level scale (range 1–9) [37], Charlson Comorbidity Score [35], a ‘Medical severity’ score (range 1–3) [41] and the percentage of patients presenting with severe comorbidity [43]. The most common treated medical conditions in relation to HaH were urinary tract infection, pneumonia, infectious disease, dehydration, and acute exacerbation of COPD. Comorbidity of patients was described as ‘several previous chronic conditions and medication’ [41] or ‘…(COPD)’ [37] or ‘significant comorbidity’ [36].

**TABLE 4:** Patient characteristics and causes of referral (*n* = 10).

#	Age	% Female	Comorbidity or general severity of disease	Causes of referral/Reason for admission
Cramer	> 18 years	55.2% female	Charlson Comorbidity Score; 0: 218 patients 1: 62 patients ≥ 2: 99 patients	Not described
Mogensen	84 years (median, all patients)	64% female	Significant comorbidity	UTI Electrolyte disturbances Dehydration Fever Acute exacerbation of COPD Pneumonia Delirium
Udesen	80 years (median)	52.3% female	Not described	Infections Clinical assessments (34.2%)
Bove	50% > 80 years	Not described	Patients admitted to cross‐sectoral and interdisciplinary team (TST) have many different diagnoses, derived treatment and care needs	UTI Dehydration Requiring intravenous fluid therapy Pneumonia Other infections requiring intravenous treatment and/or monitoring of CRP Pain Constipation Erysipelas New medical treatment
Teske	Not described	Not described	Not described	Infections Heart failure Deep venous thrombosis Electrolyte disturbances Dehydration
Kastengren	67.5 years (median)	42% female	Patients had a *median medical severity of 2* and a majority of patients had several previous chronic conditions and medications	63 unique medical conditions. Patients with infectious disease (44%) and pulmonary disease (17%) being the most common
Smedemark	81.6 years; SD ± 8.4 (mean)	54% female	Median Clinical Frailty Scale level was 5 (range 1–9)	Infections UTI Deep vein thrombosis Dehydration Fever Cough Chest pain Functional decline Acute exacerbation of COPD Pneumonia Viral infection Suspected malignancy Orthostatic hypotension Heart problem Skin infection Anaemia Unclear but ill
Thomsen	Data not available yet	Data not available yet	Data not available yet	Patients with an acute medical condition that is based on a clinical evaluation between the referring physician and the ED physician
Falchenberg	Not described	Not described	Not described	Treatable conditions that have occurred suddenly, not requiring advanced hospital‐based resources
Erwander	84 years (mean)	No significant differences regarding sex	64% with severe comorbidity (pre‐intervention group) 66% with severe comorbidity (post‐intervention group)	Health deterioration leading to a need for MHC, for example^a^: Fever New onset of breathing difficulties Increasing/acute leg swelling Infection Fall trauma Wound injury General deterioration in condition New onset of pain

Abbreviations: COPD, chronic obstructive pulmonary disease; CRP, C‐reactive protein; ED, emergency department; UVI, urinary tract infection.

^a^ Based on mail correspondence with first author Karin Erwander.

## 4 Discussion

This scoping review included ten Scandinavian studies that described HaH organisations, interventions and patient characteristics. The ten papers described different organisations depicted with three illustrated models. The models differ according to which health care sectors are involved and whether the acute care nurses are affiliated in a municipality, a hospital or a specialised unit. Most of the studies describe the affiliation of acute care nursing teams when explaining the organisation's setup. Regardless of HaH organisation, the acute care nursing teams were required to collaborate across health care sectors with hospital physicians, general practitioners, or a combination of both, depending on the severity of the patient's illness. The most common interventions were treatment with intravenous fluid, antibiotics, measurement and monitoring of blood values and vital signs. The majority of patients offered HaH were adults ≥ 65 years referred for treatment of infection, dehydration or acute exacerbation of COPD. This discussion will explore Scandinavian HaH model variations, weighing their strengths and weaknesses for successful implementation.

### 4.1 Building Expertise: A Prerequisite for Effective HaH Implementation

In all three HaH organisation models presented in this review, specialised acute nursing teams were involved in treating patients at home. Several international studies have described the need for special competency requirements for delivering HaH services [7, 13, 44]. Implementing HaH interventions may be more effective when nurses possess specialised competencies or experience in acute care. In‐home care settings, Advanced Nurse Practitioners (ANPs) who hold postgraduate education at the master's level often play a pivotal role. Their involvement in physician‐led care has been shown to improve outcomes, particularly in symptom management, self‐care, behavioural outcomes, patient satisfaction, quality of life, and health care utilisation [44, 45, 46]. In the studies included in this review, the terms used to describe the skills and abilities of the nurses vary. The study by Mogensen et al. [36] described that the municipality nurses completed a one‐day training programme that provided sufficient skills to assess the acute patient. Otherwise, the studies describe HaH nurses as specialised, highly qualified and/or experienced [4, 47, 48]. In Scandinavia, training as a registered nurse only provides generalist competencies with nurses gaining additional competencies through formal and informal education [49]. Implementing a HaH intervention must consider the competencies available and whether further training and specialisation are required.

The types of physicians delivering HaH care and treatment included municipal physicians, ED physicians, internal medicine specialists, and geriatrics specialists. ED physicians are accustomed to working with triage in acute settings, focusing on treating and stabilising life‐threatening medical conditions. In contrast, internal medicine and geriatrics specialists have a broader perspective on the complexity of medical conditions, which often characterise chronically ill patients and/or elderly fragile, comorbid patients. It was unclear which type of physician's speciality offered real value to a HaH intervention and prevented admission or readmission. Within Model 3, the unit focuses solely on HaH care provision with the physicians conducting daily digital video rounds [41]. Most often, in Model 1 and 2, a physician alone or a physician and a nurse will assess the patient in person as opposed to Model 3. Therefore, the physicians in Model 1 and 2 can diagnose and prescribe treatment more confidently as they have direct clinical patient contact, whereas the physicians from Model 3 can ensure quick diagnosis and treatment, as well as easy communication channels.

A limitation of Model 1 is the availability and accessibility of an accountable physician. Some of the derivatives of Model 1 do not have a physician who sees the patient in person [35, 36, 37, 38, 39]. Patients receiving HaH have expressed concerns about the availability of qualified professionals, specifically worrying about immediate accessibility and prompt treatment in case of deterioration [34, 50]. Furthermore, studies indicate that physician involvement plays a key role in securing patient acceptance of HaH [4, 51]. Therefore, if implementing Model 1 resources must ensure that communication channels and contingency plans for accessibility are well‐defined [4, 52].

### 4.2 The Organisations Behind HaH—Pathways for Delivering Acute Care at Home

We found that these specialised acute nursing teams had different organisational affiliations in the three HaH models. Within the organisation Model 1, the specialised acute nursing teams are affiliated with the municipality [35, 36, 37, 38, 39]. Effective communication and coordination between regular home nursing staff, acute care nursing teams and GPs or hospitals is essential for patient safety and the smooth functioning of the programme [5, 20, 51]. The close collaboration between the municipality's regular home nursing staff and the acute HaH nursing staff, which is naturally built into model 1, can provide a significant advantage for patients' safety. In Model 2, acute nurses are affiliated with the hospital [34, 40]. Model 3 organises all health professionals delivering HaH, including acute care nurses, within a single specialised hospital unit and acute care nurses typically provide the in‐person care [41]. A Cochrane review by Wallis et al. found that maintaining responsiveness and managing capacity are required to safely and effectively implement HaH [20]. In Model 1 and 2, a physician accompanies the mobile unit, whereas Model 3 offers direct video contact with a physician. In Model 1 and 3, the physician can rapidly attend the patient in person, as their affiliation is dedicated exclusively to the municipality or command centre, whereas physicians in Model 2, who were affiliated with ED, also had hospital‐based responsibilities, such as care for admitted patients resulting in potentially delayed action.

### 4.3 Adapting HaH Models to Different Population Densities and Care Contexts

Model 2 describes a mobile HaH intervention integrated within the hospital providing services for a wide catchment population, which can raise questions about the cost‐effectiveness. Mobile care may become less cost‐effective if patients' homes are far apart, leading to a low patient load per shift. A home visit by a mobile team should cost less than a day of hospital care, but more research is needed before it can be confidently stated that mobile care is more cost‐effective than hospital care [40]. Implementing Model 2 may be more appropriate in larger cities compared with models prioritising acute nursing teams affiliated with a municipality (e.g., Model 1) [34]. Although implementing Model 2 in a densely populated area may seem practical and resource‐efficient, a mobile HaH solution could also be beneficial for patients with limited geographical access to health care. While the intention is to bring care closer to citizens, the critical concern is cost implications.

The safety and efficacy of Model 3 with remote physician consultation for acutely ill patients is unclear [53]. If the model is applied to geographically dispersed populations, it can be particularly resource‐intensive, as long travel is time consuming and limits the number of patients that can be reached by acute care nurses daily [53]. In contrast, implementing the model in a densely populated area may be more cost‐effective. In Kastengren's study [41], the HaH unit is based in capital Stockholm, Sweden suggesting greater feasibility, whereas Teske's study [40] describes HaH mobile care across south‐eastern Sweden, where longer distances between patients make implementation more resource‐demanding.

Most of the studies in the review focused on interventions such as administering intravenous fluids and medications, conducting blood tests and measuring vital signs. None of the studies reported adverse events or infections related to home‐based treatments. However, patient safety is a critical concern in any health care setting, and it becomes particularly complex in the context of HaH. While HaH offers many benefits, such as reduced hospital congestion, increased patient satisfaction and the potential for faster recovery [5], it also raises important questions about ensuring patient safety outside the controlled environment of a hospital and whether there are relatives nearby who are willing to step in. Patient safety must remain a top priority. Rigorous protocols, adherence to national guidelines that describe best practice, appropriate technologies, trained health care providers, monitoring, evaluation and strong communication networks must be in place to safeguard the well‐being of HaH patients [54]. Key considerations should include monitoring and supervision as well as training and competency of health care professionals [55]. Ensuring proper infection prevention and control practices is vital to avoid infections from the home environment, where standard hospital protocols may be more challenging to implement [48].

Patients admitted to HaH had various diagnoses, and we could not conclude which specific patient categories may benefit from HaH rather than standard hospital treatment. Only four out of ten studies described the severity of the patient's condition [36, 37, 41, 43]. However, it can be expected that patients with multimorbidity treated at home are at a higher risk of hospital readmission than non‐chronic patients, which should be considered when evaluating outcome measures such as readmission and mortality.

### 4.4 Limitations and Strengths

One limitation of this study was the narrow interpretation of the HaH concept. Many countries consider HaH as any form of home‐based mobile care for any form of acute patients. In this study, HaH refers to care accompanied by follow‐up, distinguishing it from care that does not include follow‐up, for example ambulance services or prehospitalisation mobile units. However, many studies focusing on mobile care insufficiently describe the details of patient follow‐up. So, it remains unclear whether the intervention consists solely of a single contact with a patient or whether mobile care includes follow‐up visits by hospital‐ or municipality‐based physicians or nurses. For inclusion in this review, we required explicit descriptions of follow‐up, as isolated single contacts were excluded from our HaH definition. Nevertheless, studies describing mobile care are critically relevant in the broader context of reducing hospital admissions.

A notable gap identified in this review is the role of GPs in HaH models. Most GPs lack the training and experience to provide hospital‐level acute care, and the available models provide very limited detail on how they are integrated safely. While one model describes the involvement of municipal GPs, it remains unclear how such arrangements operate in practice. This highlights an important area for future research on how GPs can be effectively supported and incorporated within HaH models.

Another limitation of this review is that the Scandinavian regions included are not homogeneous in terms of health care organisation, which may affect comparability. Across all countries, there is no overarching national implementation of HaH models; models remain locally or regionally managed and vary in scope, staffing, and eligibility criteria. These structural and operational differences limit the generalisability of direct comparisons and highlight the importance of contextual interpretation when assessing HaH organisation across settings.

A strength of this study was the rigorous adherence to established review guidelines [22] and the implementation of a double‐blinded screening and extraction. Additionally, feedback from independent reviewers on the protocol article significantly enhanced the quality of the review. The first author's professional background in nursing and acute care contributed valuable expertise, offering a nuanced understanding of organisational structures in Scandinavia. This implicit knowledge was important in designing and analysing the various models examined.

## 5 Conclusion

Scandinavian HaH can be organised in three models: Multi‐affiliated health care professionals from different organisations (Model 1), hospital‐affiliated health care professionals (Model 2), or a dedicated command centre unit (Model 3) with care delivered by mobile teams in all models. Each model describes different collaboration between municipalities, GPs and hospitals, but the models are particularly distinguished by the different affiliations of the nursing teams. The review, through a depiction of three models, demonstrates the different approaches to HaH in Scandinavia, illustrating the influence of municipal, regional and national structures on care organisation.

## Author Contributions

The review aim and design were developed by K.K.H., C.M., and M.E., and refined through discussion with all co‐authors. K.K.H. and C.M. carried out the literature search and article screening in collaboration with D.E.H. and P.L.V. Data analysis and interpretation of the findings was carried out by K.K.H., C.M., and M.E. K.K.H. and C.M. prepared the first draft of the manuscript. All authors reviewed and approved the final version.

## Funding

The authors have nothing to report.

## Ethics Statement

This review does not fall within the scope of activities requiring ethical approval.

## Consent

Patient consent was not required for this study as it is a scoping review based solely on previously published literature.

## Conflicts of Interest

The authors declare no conflicts of interest.

## Supporting information


**Data S1:** PRISMA‐ScR checklist.

**Table S1:** Search string for Medline.

**Table S2:** Grey literature search.

## Data Availability

The data supporting the findings of this review can be obtained from the corresponding author upon a reasonable request.
