# Supplementary material for: Mapping Scandinavian Hospital‐at‐Home Organisations and Interventions for Patients With Acute Somatic Illness—A Scoping Review
Source: Scand J Caring Sci. 2026 Sep 7;40(3):e70312. doi: 10.1111/scs.70312 (PMC13548793; doi:10.1111/scs.70312)
Supplement: Supplementary file 2 [file SCS-40-0-s002.docx]

**Supplementary Table S1 - Search string for Medline**

| **Medline (Ovid) 11.08.2025** | |
| --- | --- |
| 1 | exp Home Care Services/ |
| 2 | exp Hospitals/ |
| 3 | exp Hospitalization/ |
| 4 | 2 or 3 |
| 5 | Hospital to Home Transition/ |
| 6 | 1 and 4 |
| 7 | 5 or 6 |
| 8 | (hospital* adj3 home).ti,ab,kw. |
| 9 | ((homecare or home care or home-care) and (medicalservice* or medical-service* or medical service* or health service* or health-service* or healthservice*)).ti,ab,kw. |
| 10 | ((homebased or home-based or home based) adj (medicalcare or medical-care or medical care or medicalservice* or medical service* or medical-service* or healthservice* or health-service* or health service* or healthcare or health-care or health care or careservice* or care-service* or care service* or careprogram* or care program* or care-program*)).ti,ab,kw. |
| 11 | (homeward* or home-ward* or home ward*).ti,ab,kw. |
| 12 | ((homebased or home-based or home based) and program*).ti,ab,kw. |
| 13 | admission avoidance.ti,ab,kw. |
| 14 | avoid* admission*.ti,ab,kw |
| 15 | (hospital* adj2 avoid*).ti,ab,kw. |
| 16 | (hospital* or unit* or ward* or institution*).ti,ab,kw. |
| 17 | 1 and 16 |
| 18 | ((Homebased or home-based or home based) adj2 (hospitalbased or hospital-based or hospital based)).ti,ab,kw. |
| 19 | (Homehospitali?ation* or Home hospitali?ation* or Home-hospitali?ation*).ti,ab,kw. |
| 20 | Virtual ward*.ti,ab,kw. |
| 21 | Home treatment*.ti,ab,kw. |
| 22 | ((Hospitalbased or hospital based or hospital-based) and home*).ti,ab,kw. |
| 23 | Domiciliary hospitali?ation.ti,ab,kw. |
| 24 | Advanced care at home.ti,ab,kw. |
| 25 | (Substitute adj3 inpatient*).ti,ab,kw. |
| 26 | "hospital at home".ti,ab,kw. |
| 27 | outpatient care.ti,ab,kw. |
| 28 | outpatient treatment*.ti,ab,kw. |
| 29 | 7 or 8 or 9 or 10 or 11 or 12 or 13 or 14 or 15 or 17 or 18 or 19 or 20 or 21 or 22 or 23 or 24 or 25 or 26 or 27 or 28 |
| 30 | exp Emergency Medical Services/ or exp Emergency Service, Hospital/ |
| 31 | exp Emergency Treatment/ |
| 32 | emergency department*.ti,ab,kw. |
| 33 | Emergency care.ti,ab,kw. |
| 34 | rapid response.ti,ab,kw. |
| 35 | ((sameday or same day or same-day) and emergency care).ti,ab,kw. |
| 36 | emergency health service*.ti,ab,kw. |
| 37 | emergency treatment*.ti,ab,kw. |
| 38 | emergency service*.ti,ab,kw. |
| 39 | emergency patient*.ti,ab,kw. |
| 40 | exacerbat*.ti,ab,kw. |
| 41 | (acute* adj2 illness*).ti,ab,kw. |
| 42 | (acute* adj2 assessment*).ti,ab,kw. |
| 43 | (acute* adj2 service*).ti,ab,kw. |
| 44 | (acute* adj2 decompensat*).ti,ab,kw. |
| 45 | acute medical.ti,ab,kw. |
| 46 | acute disease*.ti,ab,kw. |
| 47 | 30 or 31 or 32 or 33 or 34 or 35 or 36 or 37 or 38 or 39 or 40 or 41 or 42 or 43 or 44 or 45 or 46 |
| 48 | exp "Scandinavian and Nordic Countries"/ |
| 49 | Greenland/ |
| 50 | (denmark or danish or faeroe island* or faroe island* or finland or finnish or greenland* or iceland or icelandic or icelander* or norway or norwegian* or sweden or swedish or swede* or scandinavia* or nordic countries or nordic country or Northern Europe*).ti,ab,kw. |
| 51 | 48 or 49 or 50 |
|  | 29 and 47 and 51 |
