# Supplementary material for: Mapping Scandinavian Hospital‐at‐Home Organisations and Interventions for Patients With Acute Somatic Illness—A Scoping Review
Source: Scand J Caring Sci. 2026 Sep 7;40(3):e70312. doi: 10.1111/scs.70312 (PMC13548793; doi:10.1111/scs.70312)
Supplement: Supplementary file 3 [file SCS-40-0-s003.docx]

**Supplementary Table S2 - Grey literature search**

The used search terms were translated using Google translate combined with ChatGPT.

The prompt used for ChatGPT were the following: *Come with suggestions for search terms for "hospital at home' in the original language for the following countries: Denmark, Faroe Islands, Finland, Greenland, Iceland, Norway and Sweden.*

We excluded terms focusing on telemedicine and nursing.

| **Country** | **Website** | **Search terms** | **Date searched** | **Number of results** | **Included material^[1]^** |
| --- | --- | --- | --- | --- | --- |
| Denmark | https://www.sst.dk/da/ | Hjemmebehandling | 22 October 2024 | 9 | 0 |
| Denmark | https://www.sst.dk/da/ | hospitalsbehandling i hjemmet  (restriction: reports) | 22 October 2024 | 39 |  |
| Denmark | https://www.sst.dk/da/ | Hjemmehospital | 22 October 2024 | 0 | 0 |
| Denmark | https://www.sst.dk/da/ | Udgående hospitalsbehandling  (restriction: reports) | 22 October 2024 | 13 | 0 |
| Denmark | https://www.sst.dk/da/ | Hospital at home  (restriction: reports) | 22 October 2024 | 24 | 0 |

| **Country** | **Website** | **Search terms** | **Date searched** | **Number of results** | **Included material** |
| --- | --- | --- | --- | --- | --- |
| **Faroe Islands** | https://www.sjukrahus.fo/en | Heimabehandling | 22 October 2024 | 0 | 0 |
| **Faroe Islands** | https://www.sjukrahus.fo/en | Heimaviðgerð | 22 October 2024 | 0 | 0 |
| **Faroe Islands** | https://www.sjukrahus.fo/en | Sjúkrahúsheimi | 22 October 2024 | 0 | 0 |
| **Faroe Islands** | https://www.sjukrahus.fo/en | Útset sjúkrahúsviðgerð | 22 October 2024 | 1 | 0 |
| **Faroe Islands** | https://www.sjukrahus.fo/en | Hospital at home | 22 October 2024 | 13 | 0 |

| **Country** | **Website** | **Search terms** | **Date searched** | **Number of results** | **Included material**^[2]^ |
| --- | --- | --- | --- | --- | --- |
| Finland | https://thl.fi/etusivu | Sairaalahoito kotona | 29 October 2024 | 82 | 0 |
| Finland | https://thl.fi/etusivu | Kotisairaala | 29 October 2024 | 24 | 0 |
| Finland | https://thl.fi/etusivu | Erikoistunut kotihoito | 29 October 2024 | 8 | 0 |
| Finland | https://thl.fi/etusivu | Etäsairaanhoito | 29 October 2024 | 0 | 0 |
| Finland | https://thl.fi/etusivu | Hospital at home | 29 October 2024 | 9 | 0 |

| **Country** | **Website** | **Search terms** | **Date searched** | **Number of results** | **Included material** |
| --- | --- | --- | --- | --- | --- |
| Greenland | https://paarisa.gl/ | **Peqqinnissaqarfik angerlarsimaffimmi** | 27 October 2024 | 0 | 0 |
| Greenland | https://paarisa.gl/ | Angerlarsimaffimmi napparsimmavik | 27 October 2024 | 0 | 0 |
| Greenland | https://paarisa.gl/ | Hjemmebehandling | 27 October 2024 | 0 | 0 |
| Greenland | https://paarisa.gl/ | Behandling hjemme | 27 October 2024 | 0 | 0 |
| Greenland | https://paarisa.gl/ | Hjemme hospital | 27 October 2024 | 0 | 0 |
| Greenland | https://paarisa.gl/ | Hospital at home | 27 October 2024 | 0 | 0 |

| **Country** | **Website** | **Search terms** | **Date searched** | **Number of results** | **Included material** |
| --- | --- | --- | --- | --- | --- |
| Iceland | https://island.is/en/o/directorate-of-health | Heimasjúkrahús | 27 October 2024 | 0 | 0 |
| Iceland | https://island.is/en/o/directorate-of-health | Heimilismeðferð | 27 October 2024 | 0 | 0 |
| Iceland | https://island.is/en/o/directorate-of-health | Heilbrigðisþjónusta heima | 27 October 2024 | 0 | 0 |
| Iceland | https://island.is/en/o/directorate-of-health | Sérhæfð heimasjúkrahúsmeðferð | 27 October 2024 | 0 | 0 |
| Iceland | https://island.is/en/o/directorate-of-health | Hospital at home | 27 October 2024 | 196 | 2^[3]^^[4]^ |

| **Country** | **Website** | **Search terms** | **Date searched** | **Number of results** | **Included material** |
| --- | --- | --- | --- | --- | --- |
| Norway | https://www.helsedirektoratet.no/ | Sykehusbehandling | 27 October 2024 | 81 | <https://www.helsedirektoratet.no/tema/digital-hjemmeoppfolging-hjemmesykehus-og-velferdsteknologi/hjemmesykehus> |
| Norway | https://www.helsedirektoratet.no/ | Hjemmesykehus | 27 October 2024 | 61 | <https://www.helsedirektoratet.no/tema/digital-hjemmeoppfolging-hjemmesykehus-og-velferdsteknologi/hjemmesykehus>  <https://www.helsedirektoratet.no/tema/digital-hjemmeoppfolging-hjemmesykehus-og-velferdsteknologi/kunnskap-gevinster-og-erfaring/kartleggingsoppdrag-flytte-tjenester-hjem-rapport/_/attachment/inline/5bdae16e-f3f3-451d-a398-4bb7e2b4ffcf:7f80bfa9480ffdcfbd39f762ade2cc5550b17727/Kartleggingsoppdrag%20-%20Flytte%20tjenester%20hjem%20-%20Rapport.pdf> |
| Norway | https://www.helsedirektoratet.no/ | Fjernbehandling | 27 October 2024 | 2 | 0 |
| Norway | https://www.helsedirektoratet.no/ | Hospital at home | 27 October 2024 | 51 | <https://www.helsedirektoratet.no/tema/nasjonale-tjenester-i-spesialisthelsetjenesten/oversikt-over-nasjonale-tjenester-i-spesialisthelsetjenesten> |

| **Country** | **Website** | **Search terms** | **Date searched** | **Number of results** | **Included material** |
| --- | --- | --- | --- | --- | --- |
| Sweden | https://www.folkhalsomyndigheten.se/ | **Sjukhusvård i hemmet** | 27 October 2024 | 36 | 0 |
| Sweden | https://www.folkhalsomyndigheten.se/ | **Hemsjukhus** | 27 October 2024 | 0 | 0 |
| Sweden | https://www.folkhalsomyndigheten.se/ | **Hospital at home** | 27 October 2024 | 45 | 0 |

1. The most relevant documents from the Danish Health Authority were evaluations about acute psychiatric care teams. However, these were excluded due to the focus on psychiatric disorders.
2. Only reports about topics such as children's accidents at home, guidance on healthcare including infection prevention and elderly care, and COVID-19.
3. <https://island.is/en/news/hsu-lecture-at-innovation-forum>
4. <https://island.is/en/healthcare-services-for-the-elderly> --> under –emergency services 🡪 ‘SELMA’
